# Research progress on the predictive value of electrocardiographic indicators in the diagnosis and prognosis of children with vasovagal syncope

**DOI:** 10.3389/fcvm.2022.916770

**Published:** 2022-07-22

**Authors:** Ting Zhao, Shuo Wang, Miao Wang, Hong Cai, Yuwen Wang, Yi Xu, Runmei Zou, Cheng Wang

**Affiliations:** ^1^Department of Pediatric Cardiovasology, Children’s Medical Center, The Second Xiangya Hospital, Central South University, Changsha, China; ^2^Department of Neonatology, Xiangya Hospital, Central South University, Changsha, China

**Keywords:** vasovagal syncope, diagnosis, prognosis, electrocardiography, children

## Abstract

Neurally mediated syncope (NMS) is a common type of syncope in children in clinical practice, among which vasovagal syncope (VVS) is the most frequent. In recent years, more and more studies have been carried out to assess the diagnosis and prognosis of VVS. The electrocardiographic indicators such as heart rate variability (HRV), QT dispersion (QTd), P-wave dispersion (Pd), ventricular late potentials (VLP), deceleration ability of heart rate (DC), etc., are easy to obtain and inexpensive. With the help of electrocardiographic indicators, the diagnostic procedure and individualized treatment strategies of pediatric VVS can be optimized. This article reviews the value of electrocardiographic indicators in the diagnosis and prognosis of children with VVS.

## Introduction

Syncope is a transient loss of consciousness (TLOC) and the inability to maintain the posture due to transient global cerebral hypoperfusion, and its clinical characteristics are sudden occurrence, transient, and completely spontaneous recovery (1). Hu et al. (2) reported that the incidence of syncope in children and adolescents aged 2–18 years in Changsha was 17.37%, with a peak age of 16 years. The incidence of syncope in girls was higher than that of boys [31.69% (315/994 cases) vs. 26.28% (288/1096 cases), χ^2^ = 7.44, *P* < 0.05], and was higher in adolescence than the school-age children [28.85% (603/2090 cases) vs. 8.32% (136/1635 cases), χ^2^ = 243.21, *P* < 0.01] and the preschool-age children [28.85% (603/2090) vs. 2.71% (17/627), χ^2^ = 187.13, *P* < 0.01]. Neurally mediated syncope (NMS) is the most common cause of syncope, accounting for about 67% of syncope in children, and vasovagal syncope (VVS) is the most common hemodynamic type (3). According to the hemodynamic changes of the head-up tilt test (HUTT), VVS can be divided into three subtypes: vasoinhibitory vasovagal syncope (VVS-VI), cardioinhibitory vasovagal syncope (VVS-CI), and mixed vasovagal syncope (VVS-M) (4). Some children with recurrent syncope are accompanied by cardiac arrest lasting more than 3 s or severe hypotension and bradycardia, which are called malignant VVS (5, 6). The pathogenesis of VVS is still unclear. Hypovolemia, autonomic dysfunction, vasomotor dysfunction, baroreceptor reflex abnormalities, endothelial dysfunction, serotonin surges, and gut microbiota are involved in the underlying mechanism of VVS (7). At present, autonomic dysfunction is a focus of attention. It is believed that the transient abnormality of nerve reflex regulation causes decreased sympathetic tone and/or increased vagal tone, resulting in vasodilation, decreased blood pressure, and slowed heart rate, which contributes to an insufficient blood supply to the brain, causing syncope or pre-syncope to occur (8). Cardiac arrest in malignant VVS is caused by transient sinus node depression, which is caused by transiently intense vagal activity (6). For the judgment of autonomic nerve function, there are many studies on cardiac electrophysiological parameters, such as heart rate variability (HRV), QT dispersion (QTd), P-wave dispersion (Pd), ventricular late potentials (VLP), deceleration ability of heart rate (DC), etc. (7). The treatments for children with VVS mainly include non-pharmacological therapy, pharmacological therapy, and pacemaker therapy. Among these non-pharmacological therapy is the most important method, including health education, autonomic nerve function exercise, and increasing the intake of water and salt. The pharmacological therapy includes midodrine hydrochloride and metoprolol. The pacemaker therapy may be effective for children with VVS with cardiac arrest (4, 6, 9). Although VVS is a self-limiting disease and has a good prognosis, recurrent syncope attacks can seriously affect children’s physical and mental health and quality of life (10). The current accepted criteria to diagnose VVS in children comprise a combination of clinical data and clinical symptoms observed during HUTT. However, the HUTT may cause episodes of syncope or asystole, usually leading to discomfort among children and adding to their psychological loads, and its widespread clinical application is thus restricted (11, 12). Therefore, novel, acceptable, safe, and simple approaches are required to optimize diagnostic procedures and individualized treatment strategies for VVS in children. Based on the electrophysiological parameters, this article reviews the prediction of different electrocardiographic indicators on the diagnosis, treatment effects, and the recurrence of VVS.

## Heart rate variability in the prediction of diagnosis of vasovagal syncope in children

The variation over time of the period between consecutive heartbeats, HRV, reflects the regulation of the cardiovascular system by neurohumoral factors. Increased sympathetic nerve activity or decreased vagal nerve activity leads to an increase in the heart rate and a decrease in the HRV. On the contrary, decreased sympathetic nerve activity or increased vagal nerve activity leads to a decrease in the heart rate and an increase in the HRV (13). Therefore, HRV is often used clinically to evaluate the changes in the autonomic nervous system function. HRV includes time-domain indicators and frequency-domain indicators. In the time-domain indicators, the standard deviation of all normal-to-normal intervals (SDNN) reflects the overall tension of the autonomic nerve, the standard deviation of the average normal-to-normal intervals (SDANN), and the standard deviation of all normal-to-normal intervals index (SDNN index) reflect the sympathetic nerve activity, which is related to the slow change components of heart rate and appears to decrease with increased sympathetic nerve activity. The root mean square of differences between successive RR intervals (RMSSD) and the number of successive difference of intervals, that differs by more than 50 ms and is expressed as a percentage of the total number of electrocardiogram (ECG) cycles analyzed (pNN50), reflect the vagal nerve activity, which is related to the rapid change components of heart rate and appears to decrease with decreased vagal nerve activity. In the frequency domain indicators, total power (TP) reflects the overall tension of the autonomic nerves, ultra-low frequency (ULF), very low-frequency (VLF), low-frequency (LF), and normalized LF (nLF) reflect sympathetic nerve activity, and high-frequency (HF) and normalized HF (nHF) reflect vagal nerve activity, and the LF/HF ratio reflects the balance between autonomic nerves (14, 15).

### Head-up tilt test and heart rate variability

The HUTT is often used to diagnose unexplained syncope and clarify the hemodynamic classification, mainly to simulate orthostatic stress state in the laboratory (16). Alehan et al. (17) reported that 49 children diagnosed with VVS had differences in HRV indicators during HUTT. The nLF and LF/HF in the positive HUTT group were higher than those in the negative HUTT group in the upright tilt state [nLF: (67.2 ± 17.5) nU vs. (56.4 ± 12.3) nU, *P* = 0.01; LF/HF: (3.7 ± 2.9) vs. (1.7 ± 1.0), *P* < 0.01], whereas nHF was lower [(26.6 ± 13.4) nU vs. (39.0 ± 12.3) nU, *P* < 0.01]. The nLF and LF/HF of children with positive HUTT responses were significantly increased in the first 5 min after tilting compared with those in the supine position [nLF: (67.2 ± 17.5) nU vs. (43.9 ± 14.3) nU, *P* < 0.01; LF/HF: (3.7 ± 2.9) vs. (1.1 ± 0.8), *P* < 0.01], while nHF significantly decreased [(26.6 ± 13.4) nU vs. (48.5 ± 14.6) nU, *P* < 0.01]. Children with negative responses to HUTT also had the same changes in the first 5 min of tilting. The nLF and LF/HF were significantly increased compared to the supine position [nLF: (56.4 ± 12.3) nU vs. (39.9 ± 14.6) nU, *P* < 0.01; LF/HF:(1.7 ± 1.0) vs. (1.0 ± 0.7), *P* < 0.01] and nHF decreased significantly [(39.0 ± 12.3) nU vs. (50.4 ± 15.5) nU, *P* < 0.01]. These differences suggest that children with VVS mainly show increased sympathetic nerve activity in the supine position, and the increase in sympathetic nerve activity is more pronounced in children with positive HUTT responses than children with negative responses within the first 5 min after changing body positions, such as leaning upright. It was recommended that LF/HF > 2.7 within the first 5 min after changing body positions can be used as an indicator to distinguish positive HUTT from negative HUTT in children with VVS. The sensitivity, specificity, positive predictive value, and negative predictive value were 52%, 93%, 85%, and 41%, respectively. Evrengul et al. (18) also reported the same results that LF and LF/HF within 5 min before tilting in children with unexplained syncope were significantly higher than those of healthy children [LF:(61.1 ± 6.4) ms^2^ vs. (52.8 ± 6.0) ms^2^, *P* < 0.01; LF/HF:(1.88 ± 0.2) vs. (1.42 ± 0.1), *P* < 0.05], while HF was significantly lower [(36.1 ± 7.2) ms^2^ vs. (44.8 ± 6.4) ms^2^, *P* < 0.05]. On the other hand, LF and LF/HF were decreased significantly during syncope attacks [LF: (37.6 ± 4.8) ms^2^ vs. (58.7 ± 8.9) ms^2^, *P* < 0.01; LF/HF: (0.65 ± 0.2) vs. (1.63 ± 0.4), *P* < 0.05], while HF was significantly increased [(58.9 ± 6.2) ms^2^ vs. (50.5 ± 2.0) ms^2^, *P* < 0.05], and all of these indicated that the basic sympathetic nerve activity in children with unknown syncope was decreased before the syncope attack, but the vagal nerve activity was increased during the syncope attack. However, further statistical differences in these indicators were not observed in different hemodynamic types of VVS. Ciliberti et al. (19) observed that there was a significant statistical difference in VLF between the syncope and non-syncope groups in adults in the supine position (2421.09 ms^2^ vs. 895.49 ms^2^, *P* < 0.01). According to the receiver operating characteristic (ROC) curve (AUC = 0.889), VLF > 2,048 ms^2^ was recommended as the best cutoff value for predicting syncope during HUTT, and its sensitivity, specificity, positive predictive value, and negative predictive value were 87.5%, 72.2%, 75%, and 89%, respectively. However, it remains controversial whether VLF is also statistically different in children. Chen et al. (20) found that VLF had a difference between NMS children and healthy children [(34668.56 ± 17039.12) Hz vs. (25391.12 ± 11040.34) Hz, *t* = 2.025, *P* < 0.05]. However, Alehan et al. (17) and Shim et al. (21) did not find a statistically significant difference in VLF between VVS children and healthy children.

### Different hemodynamic types and heart rate variability

Different hemodynamic types of VVS were also associated with HRV. Zygmunt et al. (22) observed that the SDNN of VVS-CI was significantly lower than that of VVS-M children (114 ms vs. 164 ms, *P* < 0.05), suggesting that different hemodynamics may have different autonomic nerve function sensitivities. Guzmán et al. (23) found that the HF of VVS-CI and VVS-M in the supine position were significantly higher than those of VVS-VI children [(10.52 ± 0.48) ln(ms^2^) vs. (7.56 ± 1.3) ln(ms^2^), (11.09 ± 1.9) ln(ms^2^) vs. (7.56 ± 1.3) ln(ms^2^), *P* < 0.01, respectively]. Also, during the entire tilting process, the HF in the VVS-CI and VVS-M groups showed a downward trend until the syncope occurred 1 min later, suggesting that the pathogenesis of VVS-CI and VVS-M may be due to the increase in the vagal nerve activity. While the RMSSD and pNN50 of the VVS-VI children were significantly decreased [RMSSD: (18 ± 7) ms vs. (52 ± 17) ms, (18 ± 7) ms vs. (81 ± 9) ms, *P* < 0.01, respectively; pNN50: (2 ± 2)% vs. (20 ± 10)%, (2 ± 2)% vs. (25 ± 14)%, *P* < 0.01, respectively], suggesting that the pathogenesis of VVS-VI is related to the predominance of sympathetic nerve activity. Wang et al. (24) observed 85 syncope children aged 7–16 years and found that the daytime ULF (dULF), nocturnal ULF (nULF), daytime VLF (dVLF), and nocturnal VLF (nVLF) of children with VVS were significantly higher than those children with postural tachycardia syndrome (POTS). The dULF cut-off value is 36.2 ms^2^ (AUC = 0.826), with a sensitivity of 73.3% and a specificity of 72.5% in distinguishing VVS from POTS.

The above HRV indicators in children with VVS have certain statistical differences in different hemodynamic types during HUTT. VLF > 993.52 ms^2^ (AUC = 0.645) and dULF>36.2 ms^2^ can be recommended for the initial diagnosis of patients with VVS. For the diagnosis of different hemodynamic types, further research is needed to explore the relationship between the HRV and children’s diagnosis and the prediction of efficacy.

## QT dispersion in the prediction of diagnosis of different hemodynamic vasovagal syncope and individualized therapy

The QT interval on the ECG represents the time from the beginning of ventricular depolarization (beginning of the QRS complex) to the end of ventricular repolarization (end of the T wave). It is affected by the heart rate and the autonomic nerve, and is shortened by increased sympathetic nerve activity and prolonged by increased vagal never activity. Prolongation of the QT interval has been associated with arrhythmias and sudden cardiac death in several conditions, but particularly the inherited long QT syndrome (LQTS) (25–27). QTd refers to the difference between the maximum QT interval (QTmax) and the minimum QT interval (QTmin) in the ECG, which mainly reflects the inhomogeneity of ventricular muscle repolarization. It increased with the imbalance between the sympathetic nerve and the vagal nerve. QTd can be used as an indicator to predict malignant ventricular arrhythmias, sudden cardiac death, or syncope, and is often associated with arrhythmic events (such as LQTS, heart failure, coronary artery disease, post-myocardial infarction, or hypertrophic cardiomyopathy) (26, 27). Since the QT interval is affected by heart rate, corrected QT interval (QTc interval), and corrected QTd (QTcd) are often used (28). For children with VVS who have QT interval changes, it is necessary to be alert to the occurrence of arrhythmia events and the risk of sudden death. Kula et al. (29) observed syncopal children aged 8–18 and found that there was a difference in QTcd circadian rhythms between children with positive responses to HUTT, negative responses to HUTT, and healthy children. Compared with the negative HUTT group and healthy children, the QTcd in the positive HUTT group was significantly higher in the morning and night. [07:00–09:00: (76.92 ± 4.31) ms vs. (63.51 ± 4.76) ms, (76.92 ± 4.31) ms vs. (53.21 ± 4.29) ms, *P* < 0.05, respectively; 23:00–01:00: (81.84 ± 1.51) ms vs. (60.03 ± 4.91) ms, (81.84 ± 1.51) ms vs. (57.79 ± 7.0) ms, *P* < 0.05, respectively]. These results are consistent with the epidemiology that VVS mostly occurs in the morning. KarataS̨ et al. (30) reported 152 children aged 4–18 with a history of syncope and 67 healthy children and found that the QTd and QTcd of the positive HUTT group were significantly higher than those of the negative HUTT group [QTd: (34.9 ± 1.4) ms vs. (27.9 ± 1.3) ms, *P* < 0.01; QTcd: (58.5 ± 2.1) ms vs. (42.5 ± 1.7) ms, *P* < 0.01], and QTd and QTcd in positive HUTT group were significantly higher than those of healthy children [QTd: (34.9 ± 1.4) ms vs. (25.8 ± 1.0) ms, *P* < 0.01; QTcd: (58.5 ± 2.1) ms vs. (37.2 ± 1.4) ms, *P* < 0.01]. Also, it was recommended to use QTcd > 50 ms as an indicator for predicting positive responses to HUTT, with a specificity and sensitivity of 76.5% and 59.5%, respectively. In this experiment, the positive HUTT group included two subtypes, VVS and POTS, and no statistical difference was found in QTcd between the two subtypes. Khalilian et al. (31) also found that the QTd in the positive HUTT group was significantly higher than that of the negative HUTT group [(42.37 ± 9.52) ms vs. (23.85 ± 4.79) ms, *P* < 0.01], and compared with VVS-VI, the QTd of VVS-M group was significantly increased [(46.74 ± 9.64) ms vs. (38.00 ± 7.29) ms, *P* < 0.05]. According to ROC, QTd > 32 ms (ACU = 0.944) and QTd > 40 ms (AUC = 0.784) were recommended as indicators for predicting positive HUTT results and VVS-M type, respectively. The sensitivity and specificity were 92% and 98% and 84%, and 63%, respectively. It suggested that VVS-CI may be more sensitive to QTd changes than VVS-VI, but the disadvantage is that the experiment did not set up a control comparison of QTd between the VVS-CI group and other subtypes. Liu et al. (32) found that the related indicators of QT interval in the VVS-CI group were longer than those in healthy children [QTmax: (414 ± 18) ms vs. (386 ± 15) ms, *t* = –10.44, *P* < 0.01; QTmin: (379 ± 17) ms vs. (364 ± 16) ms, *t* = -5.892, *P* < 0.01; QTd: (34 ± 6) ms vs. (22 ± 6) ms, *t* = –12.504, *P* < 0.01; QTcmax: (464 ± 19) ms vs. (443 ± 19) ms, *t* = –7.086, *P* < 0.01; QTcd: (38 ± 6) ms vs. (26 ± 7) ms, *t* = –11.499, *P* < 0.01], and the follow-up after non-pharmacological therapy showed that indicators of QT interval in VVS-CI children were longer in the non-response group than in the response group [QTmax: (418 ± 13) ms vs. (402 ± 16) ms, *t* = 2.713, *P* < 0.05; QTd: (37 ± 4) ms vs. (29 ± 5) ms, *t* = 4.222, *P* < 0.01; QTcmax: (477 ± 14) ms vs. (455 ± 14) ms, *t* = 3.767, *P* < 0.01; QTcmin: (435 ± 13) ms vs. (422 ± 14) ms, *t* = 2.455, *P* < 0.05; QTcd: (42 ± 4) ms vs. (33 ± 7) ms, *t* = 3.745, *P* < 0.01]. According to ROC, QTd > 28.50 ms (AUC = 0.914) and QTd < 34.50 ms (AUC = 0.906) were recommended as indicators for the differential diagnosing of VVS-CI and estimating its prognosis, respectively. The sensitivity and specificity were 86.30% and 84.95% and 90.00% and 82.35%. Wang et al. (33) observed and followed up on 40 cases of children with POTS and found that the symptoms of those with QTcd ≥ 43.0 ms were improved after physical treatment [(69.2 ± 31.2) ms vs. (43.5 ± 25.9) ms, *t*/Z = 2.58, *P* < 0.05], indicating that QTcd ≥ 43.0 ms (AUC = 0.73) can be used as a predictor of physical treatment with a sensitivity of 90% and a specificity of 60%. Subsequently, Wang et al. (34) observed and followed up on 50 children with POTS and found that the symptoms of those with QTcd ≥ 47.9 ms were improved after metoprolol treatment [(66.3 ± 20.3) ms vs. (45.7 ± 19.9) ms, Z = -3.339, *P* < 0.01], indicating that QTcd ≥ 47.9 ms (AUC = 0.822) can be used as a predictor of the efficacy of metoprolol in the treatment of POTS, with a sensitivity of 78.9% and a specificity of 83.3%. Similar to POTS, some children with VVS can also choose metoprolol therapy or physical treatment, both of which have autonomic dysfunction or high catecholamine status. Therefore, when the QTcd ≥ 43.0 ms, physical treatment can be selected. When QTcd ≥ 47.9 ms, metoprolol treatment can be recommended. In conclusion, children with VVS have abnormal QT intervals, and there are statistical differences in QT intervals between different hemodynamic types. According to ROC, it was recommended that QTd > 40 ms (AUC = 0.784) and QTd > 28.50 ms (AUC = 0.914) can be used as indicators to predict VVS-M and VVS-CI, and QTd < 34.50 ms (AUC = 0.906) can predict that treatment of VVS-CI has a good prognosis.

## P-wave dispersion in the predicted diagnosis of cardioinhibitory vasovagal syncope

The P wave is the potential change that occurs when the left and right atria are depolarized. Autonomic dysfunction can cause changes in the amplitude, duration, and morphology of the P-wave. When the sympathetic nerve activity increases, the P-wave amplitude increases, forming a “pulmonary-type P-wave,” and the maximum P-wave duration (Pmax) and Pd are significantly increased (35). Prolonged P-wave duration (Pwd) (Pwd > 120 ms), a marker of left atrial abnormality, has been linked with electromechanical dysfunction and poor left atrial contractility, which has been associated with myocardial fibrosis, heart failure, atrial fibrillation, and sudden cardiac death (36, 37). Pd refers to the difference between Pmax and the minimum P-wave duration (Pmin) on the surface ECG. It is a sign of inhomogeneous electrical activity in the atria and an important predictor of cardiac arrhythmias, especially atrial fibrillation (35), and is associated with sudden cardiac death and severe ventricular arrhythmias (36, 38, 39). Kose et al. (40) reported that 100 cases of VVS children had significantly prolonged Pd compared with healthy children [HUTT positive: (50.2 ± 18.5) ms vs. (32.0 ± 11.2) ms, *P* < 0.05; HUTT negative: (39.6 ± 14.2) ms vs. (32.0 ± 11.2) ms, *P* < 0.05], the Pd in the positive HUTT group was also significantly longer than that of the negative HUTT group [(50.2 ± 18.5) ms vs. (39.6 ± 14.2) ms, *P* < 0.05]. In the positive HUTT group, there were also significant differences in Pd among different VVS subtypes, and the Pd in the VVS-CI group was significantly longer than that in VVS-VI and VVS-M [(51.1 ± 23.6) ms vs. (46.9 ± 21.7) ms, (51.1 ± 23.6) ms vs. (44.4 ± 15.8) ms, both *P* < 0.05, respectively]. It verified the existence of autonomic dysfunction in children with VVS, suggesting that Pd can be used as an ECG indicator for early clinical prediction of VVS, and may be more sensitive in VVS-CI type. Wang et al. (41) reported that the P-wave related parameters of 43 cases of children with VVS-CI aged 5∼17, such as Pd, Pmax, Pwd, corrected the maximum P-wave duration (Pcmax), corrected P-wave dispersion (Pcd), were significantly longer than those of healthy children [Pd: (36 ± 7) ms vs. (26 ± 4) ms, *t*/Z = 8.270, *P* < 0.01; Pmax: (97 ± 7) ms vs. (88 ± 4) ms, *t*/Z = 7.128, *P* < 0.01; Pwd: (79 ± 7) ms vs. (75 ± 4) ms, *t*/Z = 3.639, *P* < 0.01; Pcmax: (120 ± 12) ms vs. (112 ± 7) ms, *t*/Z = 3.390, *P* < 0.01; Pcd: (44 ± 8) ms vs. (33 ± 5) ms, *t*/Z = 7.043, *P* < 0.01]. It is further verified that children with VVS-CI have autonomic dysfunction and abnormal atrial electrical activity. Pd ≥ 27.42 ms (AUC = 0.908) was recommended as an ECG indicator for early clinical prediction of VVS-CI, with the sensitivity and specificity of 95.35% and 69.77%, respectively. In addition, de Gregorio et al. (42) observed 55 syncopal patients aged 14–75 years old and found that 75% of patients with P-wave peaking (PWP, percent increase in PWP from rest to both 15-min and peak-HR) ≤50% at peak heart rate had positive responses to HUTT, while only 5% of patients with PWP ≥ 100% had positive responses to HUTT, suggesting a potential relationship between HUTT positivity and low or no PWP. However, the relevant data on children have not been retrieved yet, so the PWP indicator deserves further study. Therefore, for P-wave related indicators, Pd = 27.42 ms (AUC = 0.908) is currently recommended as an ECG indicator for early clinical prediction of VVS-CI.

## T wave in the prediction of diagnosis vasovagal syncope and guide individualized treatment

T wave refers to the ventricular repolarization process on the synchronous 12-lead ECG. Since most of the repolarization potentials cancel each other during the ventricular repolarization process, the T wave actually reflects the potential difference of ventricular repolarization that has not been canceled out. Autonomic dysfunction can cause T wave changes, with an incidence of 20–40%. For example, when sympathetic nerve activity increases, T waves can appear bimodal, flat, or inverted, and the Niagara waterfall-like T wave (giant T wave inversion) may even occur when the activity of the sympathetic nerve increases excessively. When vagal nerve activity increases, the T wave height tip may appear, and T wave changes can be used as a predictor of sudden cardiac death and malignant arrhythmia (43, 44).

### T wave amplitude and morphology

VVS is related to T wave amplitude and morphology. Mayuga et al. (45) found that T wave changes in leads aVF, V5, and V6 were correlated with VVS (*P* < 0.05). Kolarczyk et al. (46) observed T wave changes in 30 children with VVS, and 19 patients had T wave morphological changes after HUTT, mainly in leads V4, V5, and V6, and during the HUTT in these 19 children, the QTc and T wave peak-to-end interval (Tp-Te interval) in lead V5 were significantly longer than the syncope children without T wave morphological changes [QTc: (451.3 ± 13.4) ms vs. (434.4 ± 15.4) ms, *P* < 0.01; Tp-Te: (100.0 ± 3.3) ms vs. (88.2 ± 4.0) ms, *P* < 0.01]. Markiewicz-Łoskot et al. (47) also found that 23 of 40 children with negative HUTT VVS had T wave morphological changes in lead V5 during HUTT, and the QTc and Tp-Te intervals were significantly longer than those of VVS children without T wave changes, and there is a risk of arrhythmia. Xue et al. (48) reported that children with unexplained syncope had lower T wave amplitudes in lead V3–V6 during HUTT than healthy children, and the positive HUTT group had lower T wave amplitudes in some leads (II, III, aVR, aVL, and aVF) during HUTT than negative HUTT group (all *P* < 0.05). Wu et al. (49) reported that the morphological changes of T wave were correlated with VVS, and the incidence of syncope can be reduced after oral β-blockers in VVS children with orthostatic T wave changes (28.6% vs. 72.7%, *P* < 0.01), suggesting that treatment of β-blocker can be recommended to VVS children with orthostatic T wave changes. Wu et al. (50) reported that the T wave amplitude in leads II, aVR, and aVF during syncope had a certain predictive value for short-term non-pharmacological therapy in children with VVS.

### T wave peak-to-end interval (Tp-Te interval) and Tp-Te/QT

The Tp-Te interval refers to the total time from the peak of the T wave to the end of the T wave on the synchronized 12-lead ECG, which represents the difference between the epicardial repolarization time and the M-cell repolarization time during the cardiac repolarization process, and is an indicator of transmural dispersion of repolarization (TDR). The prolongation of Tp-Te interval has predictive values for clinical malignant ventricular arrhythmias (51–53). Tp-Te/QT reflects the proportion of Tp-Te interval in the process of repolarization. As a more sensitive indicator for judging arrhythmia, it can eliminate the influence of heart rate and individual QT interval variability on Tp-Te (54). Markiewicz-Łoskot et al. (47) found that the Tp-Te interval in lead V5 was consistently prolonged during HUTT in 40 children with VVS compared to healthy children [in supine position: 89 ms vs. 80 ms, *P* < 0.01; syncope occurred: 100 ms vs. 60 ms, *P* < 0.01; after syncope: 90 ms vs. 80 ms, *P* < 0.01]. According to ROC, Tp-Te interval > 70 ms (AUC = 1) was recommended for predicted VVS with good sensitivity and specificity (both 100%).

The above studies show that β-blockers or non-pharmacological therapy is recommended for VVS children with T-wave morphology and T-wave amplitude changes, and the diagnosis of VVS can be preliminarily predicted when the Tp-Te interval >70 ms.

## Deceleration ability of heart rate in the prediction of vasovagal syncope diagnosis in different age groups

DC is an indicator to evaluate the function of the autonomic nervous system. Through the analysis of the RR interval in the Holter, the cardiac vagal nerve activity is quantitatively measured, that is, the decrease of DC reflects the decrease of the cardiac vagal nerve activity, and confirms that the DC damage is a strong predictor of mortality after myocardial infarction (55). Tong et al. (56) analyzed 90 cases of children’s Holter ECG and found that children with VVS had abnormal autonomic nerve function during the asymptomatic period, and it was related to age [school-age group vs. adolescence group: (7.94 ± 0.62) ms vs. (8.59 ± 1.15) ms, *t* = 2.49, *P* < 0.05]. It was recommended that the DC of school-age (7–10 years old) is 7.72 ms (AUC = 0.717), and the DC of adolescence (11–18 years old) is 8.36 ms (AUC = 0.692) as the cutoff values have a good predictive value for the initial diagnosis of VVS, and its sensitivity and specificity were 68.8%, 68.7% and 65.5%, 62.1%, respectively.

## Immediate heart rate changes to identify postural tachycardia syndrome and vasovagal syncope, and guide the application of metoprolol

Immediate heart rate change refers to the change of the patient’s heart rate from supine to an upright position, including acceleration index (AI) and 30/15 ratio. AI reflects sympathetic nervous system function, and the 30/15 ratio reflects vagal nerve system function (57, 58). Tao et al. (59, 60) observed differences in AI and 30/15 ratios between children with VVS and POTS. Compared with the POTS group, the AI of the VVS group was significantly decreased, and the 30/15 ratio increased [(23.440 ± 8.693) vs. (33.495 ± 8.472), *t*/Z = -4.724, *P* < 0.01; (1.025 ± 0.084) vs. (0.962 ± 0.067), *t*/Z = 3.187, *P* < 0.01], suggesting that the pathogenesis of VVS and POTS may be different. Also, it was found that AI and 30/15 ratio can be used as indicators to distinguish POTS and VVS, with AI = 28.180 (AUC = 0.801) or 30/15 = 1.025 (AUC = 0.738) as the cutoff value, the sensitivity and specificity of differentiating POTS from VVS were 79.2%, 73.1% or 87.5%, 61.5%, respectively. After the upright training intervention, the average acceleration index of VVS children with the improved condition was lower than those without improvement [(21.10 ± 6.61) vs. (31.36 ± 9.00), *P* < 0.01], and the predictive ability was the best when AI < 26.7 (AUC = 0.827) with sensitivity and specificity of 85.0% and 69.2%. Similarly, Zhang et al. (61) found that the heart rate before a positive response to HUTT was significantly higher in the effective treatment group with metoprolol than that of the ineffective treatment group [(123 ± 15) bpm vs. (96 ± 17) bpm, *P* < 0.01], and the heart rate increment before the positive response to HUTT showed a significant difference between the two groups [(42 ± 16) bpm vs. (18 ± 13) bpm, *P* < 0.01]. Compared with the baseline value, if an increase of 30 bpm in heart rate before a positive response to HUTT was taken as a cut-off value, with respect to predicting the metoprolol efficacy in the treatment of VVS, the sensitivity was 81% and the specificity was 80%, suggesting that β-blocker therapy is recommended for those with markedly increased heart rate before a positive response to HUTT.

## Ventricular late potential in the prediction of malignant arrhythmia in children with vasovagal syncope

VLP is a high-frequency, low-amplitude fragmented electrical activity characterized by multi-shaped sharp waves caused by local delayed depolarization of the myocardium. It is caused by slow and irregular reentry activity in the ischemic myocardium, and it is an electrophysiological indicator reflecting the instability of myocardial electrical activity (62). The main detection indicators are: (1) total QRS time (TQRS, ms), which refers to the time from the start of QRS to the end of VLP on the filtered leads X, Y, and Z; (2) root mean square value (RMS40, μV), which is the voltage at the end of the QRS wave at 40 ms; and (3) the high frequency and low amplitude time limit (LAS40, ms), the time limit when the terminal voltage of the QRS after filtering is lower than 40 μV (63). Previous studies have shown that there are circadian rhythm changes in VLP associated with autonomic nerves (64, 65), and VLP is associated with the induction of malignant arrhythmias, the prognosis of patients with myocardial infarction, and sudden cardiac death (66–68). Zhang et al. (63) found that the LAS40 of VVS children was significantly prolonged compared with healthy children [(29.04 ± 6.59) μV vs. (26.15 ± 5.82) μV, *t* = 2.204, *P* < 0.05], suggesting that VVS children have abnormal myocardial electrical activity and the risk of cardiac events, and such children should be alert to the possibility of malignant arrhythmia. Zou et al. (69) reported that the TQRS, RMS40, and LAS40 of 184 cases of VVS-VI children were longer than those of healthy children [TQRS: (84.89 ± 12.05) ms vs. (81.21 ± 8.23) ms, *P* < 0.01; RMS40: (28.73 ± 7.23) μV vs. (26.89 ± 7.36) μV, *P* < 0.05; LAS40: (62.43 ± 19.17) ms vs. (56.79 ± 18.75) ms, *P* < 0.05], and the VVS-VI group had more abnormally prolonged LAS40 (94.57% vs. 83.80%, *P* < 0.01), suggesting that monitoring VLP in children with VVS-VI can help predict the possibility of malignant arrhythmia.

## Conclusion

For the clinical prediction of VVS diagnosis (Table 1), LF/HF > 2.7, dULF>36.2 ms^2^, QTcd > 50 ms, QTd > 32 ms, Tp-Te > 70 ms, school-age DC > 7.72 ms, adolescent DC > 8.36 ms, AI < 28.18, and 30/15 > 1.025 were used as indicators to distinguish positive and negative HUTT in children, according to the maximum AUC (1.000), Tp-Te > 70 ms is recommended as a rapid identification of VVS in ECG indicator. For different hemodynamic types (Table 2), VVS-CI can be predicted with QTd > 28.5 ms and Pd ≥ 27.42 ms. According to the maximum AUC (0.914), QTd > 28.5 ms is recommended as the best ECG indicator for predicting VVS- CI, when QTd > 40 ms can be used to predict VVS-M. For the selection of VVS treatment measures and the prediction of efficacy (Table 3), QTd < 34.5 ms, AI < 26.7, heart rate before positive HUTT reaction increased by 30 bpm from basal heart rate were used as predictors for the effectiveness of non-pharmacological therapy, upright training, and metoprolol intervention.

**TABLE 1:** Predictors of the diagnosis of pediatric VVS.

References	ECG indicators	Cutoff values	AUC	Sensitivity (%)	Specificity (%)
Alehande et al. (17)	LF/HF	>2.7	–	52	93
Wang et al. (24)	dULF	>36.2ms^2^	0.826	73.3	72.5
Karatas̨ et al. (30)	QTcd	>50 ms	–	76.5	59.5
Khalilian et al. (31)	QTd	>32 ms	0.944	92	98
Markiewicz-Łoskot et al. (47)	Tp-Te	>70 ms	1	100	100
Tong et al. (56)	DC	School-age>7.72 ms	0.717	68.8	68.7
		Adolescence>8.36 ms	0.692	65.5	62.1
Tao et al. (59, 60)	AI	<28.180	0.801	79.2	73.1
	30/15	>1.025	0.738	87.5	61.5

LF/HF, low-frequency/High-frequency; AUC, area under curve; VVS, vasovagal syncope; VLF, very low-frequency; dULF, the daytime ultra-low frequency; QTcd, corrected QT dispersion; QTd, QT dispersion; Tp-Te, T wave peak-to-end interval; DC, deceleration ability of heart rate; AI, acceleration index.

**TABLE 2:** Predictors of the diagnosis of different subtypes of pediatric VVS.

References	ECG indicators	Cutoff values	AUC	Sensitivity (%)	Specificity (%)	Type
Khalilian et al. (31)	QTd	>40 ms	0.784	84	63	VVS-M
Liu et al. (32)	QTd	>28.5 ms	0.914	86.3	84.95	VVS-CI
Wang et al. (41)	Pd	≥ 27.42 ms	0.908	95.35	69.77	VVS-CI

AUC, area under curve; SDNN, the standard deviation of all normal-to-normal intervals; SDANN, the standard deviation of the average normal-to-normal intervals; SDNN index, the standard deviation of all normal-to-normal intervals index; QTd, QT dispersion; VVS-M, mixed vasovagal syncope; VVS-CI, cardioinhibitory vasovagal syncope; Pd, P wave dispersion.

**TABLE 3:** Predictors of individualized treatment of pediatric VVS.

References	Interventions	ECG indicators	Cutoff values	AUC	Sensitivity (%)	Specificity (%)
Liu et al. (32)	Non-pharmacological	QTd	<34.50 ms	0.906	90	82.35
Wu et al. (49)	β-blockers	T wave morphology	–	–	–	–
Wu et al. (50)	Non-pharmacological	T wave amplitude	–	–	–	–
Tao et al. (59, 60)	Upright training	AI	<26.7	0.827	85	69.2
Zhang et al. (61)	Metoprolol	Increment of HR before positive response in HUTT	30 bpm	–	81	80

QTd, QT dispersion; AI, acceleration index; HR, heart rate; HUTT, head-up tilt test.

VVS is a self-limiting disease caused by the disturbance of autonomic regulation, which usually occurs in early adolescence. ECG indicators such as HRV, Pd, QTd, Tp-Te interval, Tp-Te/QT, T-wave amplitude and shape, immediate heart rate change, DC and VLP are easily available, non-invasive, and inexpensive. However, there is still a lack of systematic large-scale, multi-center, long-term follow-up studies, and the value of some indicators needs to be further confirmed. With the help of relevant ECG indicators, individual treatment plans can be selected for patients with different hemodynamic types to improve the long-term prognosis of children and adolescents with VVS.

## Author contributions

TZ conceptualized, prepared, and wrote the manuscript and made the tables. SW, MW, HC, YW, YX, and RZ participated in providing documentation. RZ and CW reviewed, edited, and revised the manuscript. All authors have read and approved the final manuscript and assume full responsibility for its contents.

## Conflict of interest

The authors declare that the research was conducted in the absence of any commercial or financial relationships that could be construed as a potential conflict of interest.

## Publisher’s note

All claims expressed in this article are solely those of the authors and do not necessarily represent those of their affiliated organizations, or those of the publisher, the editors and the reviewers. Any product that may be evaluated in this article, or claim that may be made by its manufacturer, is not guaranteed or endorsed by the publisher.
